# Cytotoxicity of an Innovative Pressurised Cyclic Solid–Liquid (PCSL) Extract from *Artemisia annua*

**DOI:** 10.3390/toxins13120886

**Published:** 2021-12-11

**Authors:** Rosanna Culurciello, Andrea Bosso, Giovanni Di Fabio, Armando Zarrelli, Angela Arciello, Francesca Carella, Leonardo Leonardi, Laura Pazzaglia, Gionata De Vico, Elio Pizzo

**Affiliations:** 1 Department of Biology, University of Naples Federico II, 80126 Naples, Italy; rosanna.culurciello@unina.it (R.C.); andrea.bosso@unina.it (A.B.); francesca.carella@unina.it (F.C.); 2 Department of Chemical Sciences, University of Naples Federico II, 80126 Naples, Italy; giovanni.difabio@unina.it (G.D.F.); armando.zarrelli@unina.it (A.Z.); anarciel@unina.it (A.A.); 3 Center for Studies on Bioinspired Agro-Environmental Technology (BAT CENTER), University of Naples Federico II, 80126 Naples, Italy; 4 Department of Veterinary Medicine—Veterinary Pathology, University of Perugia, 06129 Perugia, Italy; leonardo.leonardi@unipg.it; 5 Laboratory of Experimental Oncology, IRCCS Istituto Ortopedico Rizzoli, 40136 Bologna, Italy; laura.pazzaglia@ior.it

**Keywords:** artemisinin, anticancer effects, cytotoxicity, stress granules, alternative extraction procedures, osteosarcoma cells, HeLa cells

## Abstract

Therapeutic treatments with *Artemisia annua* have a long-established tradition in various diseases due to its antibacterial, antioxidant, antiviral, anti-malaria and anti-cancer effects. However, in relation to the latter, virtually all reports focused on toxic effects of *A. annua* extracts were obtained mostly through conventional maceration methods. In the present study, an innovative extraction procedure from *A. annua*, based on pressurised cyclic solid–liquid (PCSL) extraction, resulted in the production of a new phytocomplex with enhanced anti-cancer properties. This extraction procedure generated a pressure gradient due to compressions and following decompressions, allowing to directly perform the extraction without any maceration. The toxic effects of *A. annua* PCSL extract were tested on different cells, including three cancer cell lines. The results of this study clearly indicate that the exposure of human, murine and canine cancer cells to serial dilutions of PCSL extract resulted in higher toxicity and stronger propensity to induce apoptosis than that detected by subjecting the same cells to *Artemisia* extracts obtained through canonical extraction by maceration. Collected data suggest that PCSL extract of *A. annua* could be a promising and economic new therapeutic tool to treat human and animal tumours.

## 1. Introduction

*Artemisia annua* is a member of the Asteraceae family, with therapeutic properties mainly related to its content in artemisinin, a sesquiterpene lactone produced in the trichomes, which presents an endoperoxide bridge indispensable for its bioactivities [1]. Artemisinin and its derivatives are active ingredients at the basis of most antimalarial treatments [2], as well as present intriguing anticancer properties related to their capability to arrest cell growth, interfere with the cell cycle and/or activate multiple cell death pathways in cancer cells [3,4,5]. An increasing number of evidence suggests that the anticancer properties of *A. annua* are not exclusively related to a single active component of the plant, but rather to its whole phytochemical complex, including over 600 phytochemicals dominated by sesquiterpenoids, flavonoids, coumarins, enzymes and steroids [6], which may act synergistically [7,8,9]. Studies also demonstrated that *A. annua* whole plant extracts could have more effective anticancer properties compared to single phytochemical compounds both in vitro and in vivo [10,11,12], thus supporting their promising use as new therapeutics in human and animal tumours.

However, not all the extraction methods allow to isolate from the plant fractions of active ingredients with similar properties. It is widely reported that the extraction strategies can significantly affect the potential therapeutic efficacy of obtained samples mainly due to their chemical composition [13,14,15]. In this context, an innovative technique, based on pressurized cyclic solid-liquid (PCSL) extraction, was recently demonstrated to be significantly more efficient than the traditional methodologies used to obtain mother tincture (European Pharmacopeia) in extracting phytochemicals from *A. annua* [2]. This technique is based on the creation of a pressure gradient, achieved through compressions and immediate decompressions of the plant tissue, which ultimately leads to the extraction of trichomes and avoiding any maceration. Thereby, this method reduces the operating time of extraction, avoids the use of solvents, and facilitates the use of crude extract, evading further manipulations and reducing toxicity, with high efficiency and low or no risks for the operator and the environment.

To date, the biological properties of *A. annua* PCSL extract have not been fully clarified; nevertheless, it is conceivable to suppose that the high levels of artemisinin and other phytochemicals detected in this product could have a positive impact on its anticancer properties compared to other preparations [2]. In this framework, the present study was mainly focused on assessment of the effects of *A. annua* PCSL extract, compared to canonical mother tincture, on four different cell lines: 1. Simian virus 40-transformed mouse cells (SVT2); 2. mouse embryonic fibroblast cells (NIH/3T3); 3. human cervical cancer cells (HeLa); 4. canine osteosarcoma cells (CRL2130). Collected results indicate that the phytocomplex achievable with this extraction strategy exerts significant cell toxicity, with a promising trend more accentuated on tumour cells, de facto opening the way to an alternative approach in the study of the plant extracts’ applicability.

## 2. Results

### 2.1. Cytotoxicity Tests: PCSL Extract vs. Mother Tincture of A. annua

**Figure 1:**
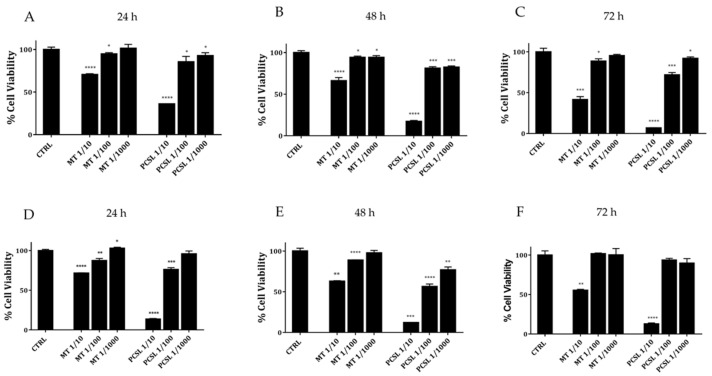
Cell viability of normal (3T3-panels **A**–**C**) and cancer (SVT2-panels **D**–**F**) murine fibroblasts treated with serial dilutions of PCSL hydroalcoholic extract (labelled as PCSL) or mother tincture (labelled as MT) from *A. annua* for 24, 48 and 72 h. Doses correspond to serial dilutions of the two different extracts obtained starting from similar masses of fresh flowering aerial part of *A. annua*. Experiments were performed in triplicate, and statistical analysis were carried out as described in Materials and Methods. A *p* value of 0.05 or less was considered statistically significant (* *p* < 0.05, ** *p* < 0.01, *** *p* < 0.001 or ****p < 0.0001).

Cytotoxicity of serial dilutions of PCSL hydroalcoholic extract and mother tincture of *A. annua* was compared by MTT assay on normal and tumour murine fibroblasts. As shown in Figure 1, data clearly highlight a marked dose-dependent toxic effect of PCSL hydroalcoholic extract on both cell lines. Moreover, in all conditions tested (scalar doses and three different incubation times), PCSL hydroalcoholic extract ever exerts a higher toxicity if compared to mother tincture of *A. annua*, thus corroborating the initial hypothesis that the PCSL extract could present new properties and higher amounts of active ingredients.

**Figure 2:**
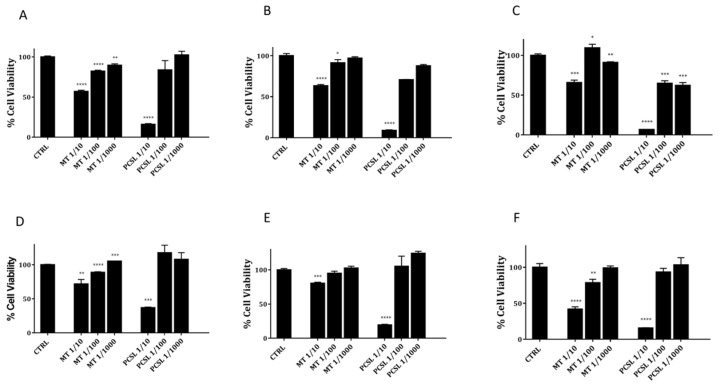
Cell viability of HeLa (Panels **A**–**C**) and CRL130 (panels **D**–**F**) treated with three different serial dilutions of PCSL hydroalcoholic extract or mother tincture extract (MT) for 24, 48 and 72 h. Experiments were performed in triplicate, and statistical analysis were carried out as described in materials and methods. A *p* value of 0.05 or less was considered statistically significant (* *p* < 0.05, ** *p* < 0.01, *** *p* < 0.001 or ****p < 0.0001).

To confirm the cytotoxic potential over time of PCSL extract, we evaluated its toxicity on further two cancer cell lines of human and canine origin (HeLa cells and canine osteosarcoma cells-CRL2130). Collected data on both cancer cell lines (see Figure 2, panels A–F) indicate a clear dose and time-dependent toxicity of PCSL hydroalcoholic extract as well as a higher toxicity if compared to that detected for similar doses from mother tincture.

### 2.2. PCSL Extract Induces Apoptosis and Negatively Affects the Recruitment of Stress Granules

**Figure 3:**
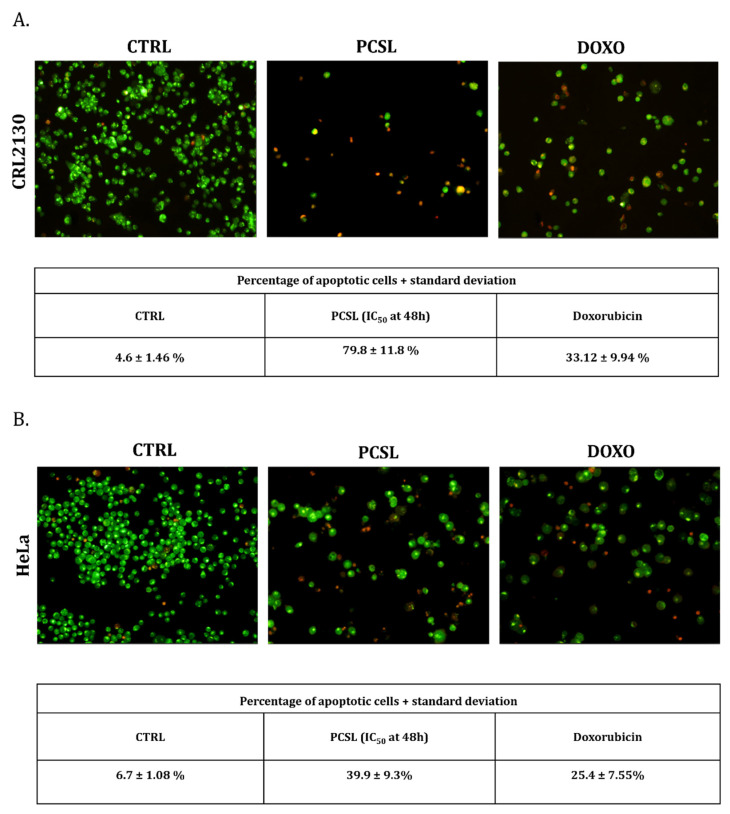
AO/EB staining of CRL2130 (**A**) and HeLa cells (**B**) treated with *A. annua* PCSL extract (doses corresponding to IC_50_ values at 48 h). Apoptotic cells are represented as red spots, while green ones indicate viable cells. Numbers below the images correspond to the means of the percentage of apoptotic cells ± SD, considering five microscopic fields for each sample.

To verify the possible PCSL extract action in the triggering of the apoptotic pathway, the differential acridine orange/ethidium bromide (AO/EB) staining was assayed on Hela and CRL2130 cells. Cells were first treated with PCSL hydroalcoholic extract (at doses equal to IC50 values determined in the MTT assays) for 48 h and then incubated with AO/EB dye mixture to detect, by fluorescence microscopy, live cells and dead apoptotic cells (see Figure 3, panels A and B). As shown in figure, both for CRL2130 (panel A) and for HeLa (panel B) treated with PCSL extracts, a significant number of apoptotic cells was detected.

**Figure 4:**
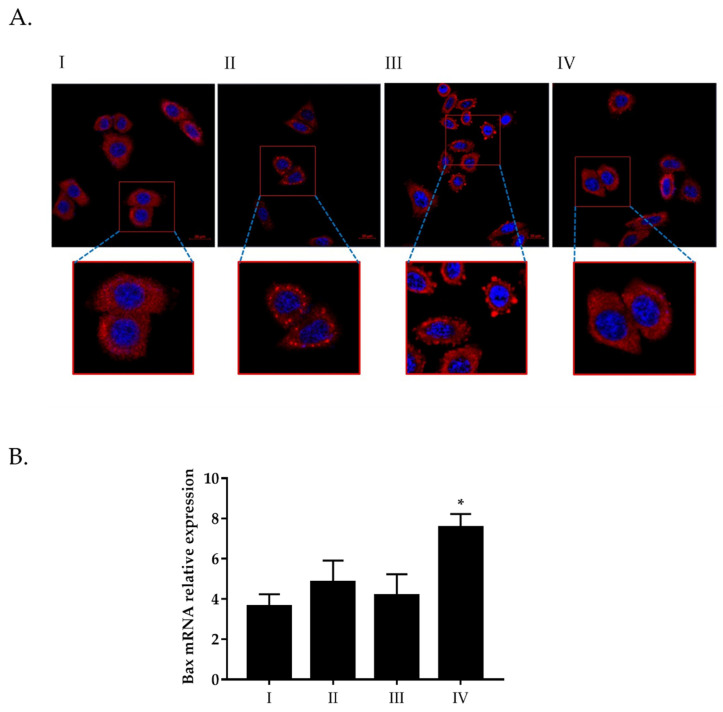
(**A**) Subcellular localization of PABP, a stress granule marker protein, in HeLa cells. (I) Untreated cells (negative control); (II) HeLa cells subjected to oxidative stress with 500 μM sodium arsenite (stress granules formation used as reference control); (III) HeLa cells treated with PCSL extract diluted 1:100 and (IV) HeLa cells treated with PCSL extract diluted 1:10. All treatments were carried out for 1 h at 37 °C. Fixed cells were stained with anti-human PABP (red), whereas nuclei were counterstained by using DAPI (blue). For each panel, an enlargement of the insert highlighted in red is shown. The bars indicate 20 μm. (**B**) Expression analysis by RT-qPCR of Bax gene. Bars represent relative expression levels of Bax gene normalized by GAPDH in the same experimental points shown above (I, II III and IV). A *p* value of 0.05 or less was considered statistically significant (* *p* < 0.05).

Based on these indications, we wanted to verify whether the action of PCSL extract could have an effect on the recruitment of stress granules, and to do this, confocal laser scanning microscopy analyses were carried out on HeLa cells pre-treated with two different dilutions of PCSL extract (see Methods). As shown in Figure 4 (panel A), the treatment with increasing doses of PCSL extract negatively affects the stress granules recruitment, both in terms of number and size, supporting the hypothesis that the toxicity of this innovative type of extract is mainly due to its ability to induce apoptosis. A further test was carried out by RT-qPCR to explore possible alterations of Bax mRNA expression, a pro-apoptotic marker. As shown in Figure 4 (panel B), a significant rise of Bax mRNA relative expression is observed at the highest concentration of PCLS extract, detected by a comparison with a housekeeping gene (GAPDH), thus suggesting the ability of this extract to affect cell viability by inducing the apoptotic pathway.

## 3. Discussion

In several reports, *A. annua* has been taken into consideration for its anti-cancer activity starting from samples obtained with canonical extraction procedures [4,16]. In the present pivotal study, we assessed the biological activity of an extract from *A. annua* obtained by an innovative technique, known as pressurized cyclic solid–liquid extraction (PCSL) [2], using two well-established in vitro models in human and animal cancer research.

There are several advantages related to the use of the Naviglio extractor as an alternative to conventional techniques: (i) the reduction of extraction times; (ii) no more than 2–24 h depending on the parts of the plant used for the extraction; (iii) the high quality of the extract, since the contact between extracted solid and liquid phases is reduced to a minimum, and therefore, the degradation of the material to be extracted is completely negligible; (iv) the obtained extract does not require filtration; (v) the negligible degradation of the extracted bioactive compounds, as the extraction process is carried out at room temperature and, if necessary, in an atmosphere of inert nitrogen; (vi) the high extraction efficiency resulting from repeated vacuum-pressure cycles [2].

The extractor applies pressure and depression cycles on the extracting liquid that is placed in contact with the solid vegetable matrix, from which the active ingredients are extracted with high efficiency, in a short time (compared to conventional maceration) and at room temperature [17]. Extracting at low temperatures is relevant in order to avoid thermal stress on different compounds. Therefore, it is possible to faithfully obtain the same composition of the substances contained in medicinal plants without inducing transformations in the bioactive constituents, which are generally the most “delicate” elements to be extracted, causing their thermolability. For this reason, the dynamic strategy of extraction, here presented, represents a promising technology useful to obtain solid matrices containing extractable compounds that are exhausted in different solvent and in their mixtures. This process is currently being used by a growing number of herbal, cosmetic, food and homeopathic companies [18,19].

Collected data highlight that the exposition of HeLa, SVT2 and CRL2130 cells to serial dilutions of extract (1:10, 1:100 and 1:1000) at three different incubation times (24, 48 and 72 h) induce strong cytotoxic effects on the cancer cells, and at the dose corresponding to dilution 1:10, this property was detectable already at short incubation times and was stable over time.

Previous studies involving HeLa cells also underlined the antineoplastic potential of *A. annua* and its active constituents (single molecules or as a whole) using canonical extraction procedures. This had an important clinical impact for the development of new therapeutic approaches in cancer treatment. Disbrow et al. [20] found that the main artemisinin derivative, dihydroartemisinin (DHA), showed a high toxicity and propensity to induce apoptosis via activation of the mitochondrial caspase pathway, in cervical cancer cells, while artemisinin had no effect [20]. Based on the results obtained on HeLa cells, Disbrow et al. tested DHA efficacy upon topical treatment of mucosal papillomavirus lesions in dogs and demonstrated a strong inhibition of viral-induced tumour formation. According to the above results, Jansen et al. [21], using an artemisinin derivative (artesunate-R), conducted a pilot study in human patients affected by cervical carcinoma. Patients treated with artesunate-R were characterized by a significant improvement of clinical manifestations and a prolonged survival time. Furthermore, observed clinical responses were associated with the gene expression downregulation of p53, EGFR, Ki-67 and CD31, which are relevant tumour proteins. Finally, a Phase I study to test the healthiness of intravaginal artesunate application in the treatment of HPV+ High Grade Cervical Intraepithelial Neoplasia is ongoing at the Sidney Kimmel Comprehensive Cancer Center at Johns Hopkins (https://clinicaltrials.gov/ct2/show/NCT02354534) (accessed on 28 September 2021) (USA).

Concerning whole extracts of *A. annua*, Efferth et al. [7] tested several of them with different phytogeographical origins and on HeLa cells. In that study, dichloromethane extract was more effective than other preparations in killing HeLa cells. Furthermore, the phytochemical composition of the extracts supported the in vitro synergistic effects of compounds that constitute the phytochemical complexes, suggesting artemisinin, arteannuin B and scopoletin as the main active components underlying the relevance of different extraction techniques on the biological effect of the extracts.

SVT2 is a malignant cell line derived from transformed murine fibroblasts and belongs to a large family of malignant cell lines transformed by SV-40, a viral agent thought to be involved in human and animal malignancy pathogenesis [22,23]. To the best of our knowledge, this is the first study on the anticancer activity of *A. annua* extract on SVT2 cells. In our study, we have compared the activity of PCSL extract on SVT2 and 3T3 cells to evaluate the selectivity of the anticancer activity of the extract. Based on obtained results, it would appear that PCSL extract from *A. annua* presents a significant dose-dependent toxicity and a detectable selectivity in an incubation time ranging from 24 to 48 h. These data reinforce the interest on PCSL extract as a promising therapeutic option, although the results should be confirmed in further research activities to optimize the dose in relation with the outcome to obtain. Among the results of our study, those obtained on CRL2130 (a canine osteosarcoma cell line) are of particular relevance. In dogs, osteosarcoma is the most common primary bone tumour that tends to occur in middle-aged specimens and in large and giant breeds, especially in the metaphyseal region of the long bones [24,25]. Canine osteosarcoma represents a relevant model in the understanding of the same disease in humans since it has genetic background, clinical symptoms, biological behaviour, treatment response and a progression comparable to that of human osteosarcoma, particularly in those occurring during adolescence [25,26]. Unfortunately, therapeutic failures are frequent in both species, with a survival time after surgery and chemotherapy only of 60% of human patients, reaching 5-year (about 20% survival time at 5 years with metastasis present at diagnosis) and two-year survival rates less than 20% in dogs (in canines with amputation and chemotherapy, the average survival time is 10–12 months) [26]. As a consequence, new therapies are desperately needed for osteosarcoma treatment to improve clinical outcome in both humans and dogs. Anti-tumour properties of artemisinin and of its derivatives have been demonstrated in both human and canine osteosarcoma cell lines [27]. Furthermore, Isani et al. [10] tested a commercial hydroalcoholic extract of *A. annua* and demonstrated that the whole phytochemical complex is more effective than artemisinin alone in killing a further canine osteosarcoma cell line (D-17). In our study, the antineoplastic activity detected for PCSL extract on canine osteosarcoma cell lines confirm that *A. annua* extract has a therapeutic potential for these tumours. In our case, a generic activation of apoptosis pathways was demonstrated in vitro after exposing cells to PCSL extract as suggested by results obtained by the differential staining assay of normal and apoptotic cells using a dye mixture with acridine orange/ethidium bromide (AO/EB) and was further supported by data of stress granules and RT-qPCR to explore possible alterations of pro-apoptotic Bax mRNA expression [28].

Stress granules are transient cytoplasmic aggregates, based on RNAs and proteins, that allow a rapid retrieval of cellular homeostasis, following stress stimuli [29]. Several reports indicate an intimate link between stress granules and cancer cells and, in particular, the ability of these complexes to inhibit trigger of apoptosis, thus integrating oncogenic signalling with the increase of cancer cell fitness [30,31]. Data of the present study show that PCSL extract negatively affects the formation of the stress granules, both in terms of number and size, strongly supporting the hypothesis that the toxicity of this innovative type of extract is mainly due to its ability to induce apoptosis. Some secondary metabolites from *A. annua*, such as scopoletin, might be responsible for the activation of a classic mitochondrial apoptosis pathway in cancer cells [32,33].

However, canine and human osteosarcomas, in line with cancer cells hallmarks, are also known to overexpress membrane transferrin receptors, which make cells particularly susceptible to a particular form of iron-dependent non-apoptotic cell death, known as Ferroptosis, the latter of which is activated by artemisinin [34]. It is, therefore, possible that the phytochemicals present in the PCSL extract of *A. annua* activate multiple molecular cell death pathways in cancer cells. Hence, further studies are necessary to clarify the nature and the number of death pathways involved in the antineoplastic activity of the extract used in our study. In conclusion, in the current focus on discovering innovative therapeutics, our preliminary results suggest that PCSL extract of *A. annua* could be a promising and economic new tool to treat animal tumours refractory to conventional agents.

## 4. Materials and Methods

### 4.1. Plant Material

Summer *A. annua* plants were harvested near Caserta (Southern Italy), characterized and deposited in the herbarium of University of Naples Federico II (HERBAZLS 280815).

### 4.2. Chemicals and Materials

Artemisinin (CAS: 63968–64–9; purity > 98%), scopoletin (CAS: 92–61–5; purity > 98%), HPLC-grade acetonitrile, methanol and phosphoric acid were purchased from Sigma Aldrich (Milan, Italy). Ultrapure quality water, generated in the laboratory using a Milli-Q water purification system (Millipore, Bedford, MA, USA), was used throughout the experiments.

### 4.3. Pressurized Cyclic Solid–Liquid (PCSL) Extractor

The Naviglio Extractor, a name that follows that of the manufacturer and the relative principle, allows to extract the compounds that are not chemically bound to a vegetable matrix with a suitable solvent, exerting a negative pressure gradient between the outside and the inside until the initial equilibrium conditions are quickly reached [17]. The extractor applies pressure and depression cycles on the extracting liquid that is placed in contact with the solid vegetable matrix, from which the active ingredients are extracted with high efficiency, in a short time (compared to conventional maceration) and at room temperature [35]. The extraction therefore takes place in conditions that do not damage the thermolabile substances, which are generally the most delicate and subject to transformation/degradation due to the effect of temperature, faithfully preserving the composition of an extract of natural origin in relation to all its active ingredients. The dynamic solid–liquid rapid extractor shows an innovative solid–liquid extraction technology that allows the solid matrices containing extractable substances to be exhausted in pure or mixed solvents with different polarity. This process is currently being used by a growing number of cosmetic, food, homeopathic and herbal companies.

### 4.4. Hydroalcoholic Extracts by PCSL Extractor

The PCSL extraction method was carried out using a Naviglio Extractor equipped with a housing of the vegetable matrix all in steel. It used ethanol:H_2_O (3:2, *v/v*, 550 mL) or H_2_O (540 mL) as extraction solvent; 45 and 35 g of fresh plant, respectively, and a pressure of about 10 bar and an immediate decompression at about 0–1 bar of pressure. An extraction cycle comprises the static and dynamic phases of 3 and 1 min, respectively. The extraction lasted 3 and 24 h in hydroalcoholic solution, respectively.

### 4.5. Qualitative-Quantitative Analysis of Artemisinin and Scopoletin by HPLC

**Figure 5:**
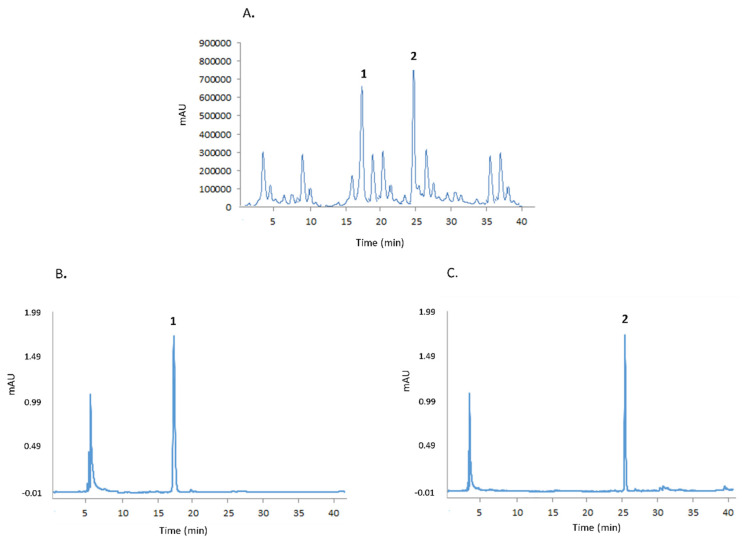
(**A**) Hydroalcoholic extract by PCSL extractor; (**B**) scopoletin (**1**); (**C**) artemisinin (**2**).

Analysis of artemisinin and scopoletin was performed using a Shimadzu LC-20 HPLC chromatograph (see Figure 5), equipped with a spectrofluorometric detector Shimadzu RF10AXL (Milan, Italy) and an analytical column RP18 (particle size 5 μm, 150 × 4.6 mm i.d., Waters, Milford, MA, USA). The amount of artemisinin and scopoletin was determined under isocratic conditions using a mixture of methanol:H_2_O:H_3_PO_4_ (50:50:0.1, *v/v/v*) and a flow of 1.5 mL/min. The excitation and emission wavelengths for artemisinin were 210 and 246 nm, respectively, while the excitation and emission wavelengths for scopoletin were 430 and 460 nm, respectively. One mL of the hydroalcoholic extract was diluted 20 times with milli-Q water, and then 100 μL of each solution was mixed with an equal volume of the mobile phase and analysed by HPLC in triplicate.

### 4.6. Preparation of Stock and Working Solutions

For the determination of scopoletin, a calibration curve was constructed using eight different solutions containing from 3 to 50 mg of standard, suitably diluting equal aliquots of a stock solution at a concentration of 1.0 mg/mL. The solvent used for the stock solution and the subsequent dilutions was ethanol, and care was taken to keep the fractions in the dark and at 4 °C before use. The prepared solutions were analysed spectrofluorimetrically against the solvent blank (absolute ethanol). For each solution, from the measurement of the fluorescence intensity (X) as a function of the quantity of standard present (Y), it was possible to obtain a calibration curve of equation equal to Y = 7.36 × 10^−5^ X, with a value of the coefficient correlation (R) equal to 0.999. The same procedure was used to obtain the calibration curve for artemisinin, starting from a stock solution with a concentration of the commercial standard equal to 1.0 mg/mL and diluted solutions with an amount of standard equal to 0.1–10 μg/mL. The line equation for the standard curve was Y = 6.11 × 10^−4^ X and correlation coefficient (R) value was 0.999.

### 4.7. Cell lines, Cell Viability Experiments and Apoptosis Assays

Simian virus 40-transformed mouse cells (SVT2), human cervical cancer cells (HeLa) and canine osteosarcoma cells (CRL2130) were used to test the anticancer activity of PCSL extract of A. annua. Mouse embryonic fibroblast cells (NIH/3T3) were used as non-tumour control cells.

SVT2 (Balb/c 3T3 mouse cells transformed by simian virus 40), NIH/3T3 (Balb/c 3T3 mouse embryonic fibroblasts), HeLa (human cervical carcinoma cells) were supplied by ATCC (www.atcc.org) (accessed on 17 November 2021). DAN CRL2130 (canine osteogenic sarcoma cells) were kindly provided by Dr. Laura Pazzaglia from Istituto Ortopedico Rizzoli (Bologna, Italy). SVT2, 3T3 and HeLa cells were maintained in DMEM supplemented with 10% FBS, 1% pen/strep and 1% L-Glu, while CRL2130 cells were cultured in EMEM supplemented with 10% FBS, 1% pen/strep and 1% L-Glu and 0.4 mg/mL G418.

To evaluate the cytotoxic potential of the PCSL extract from A. annua, 5 × 10^3^ cells were seeded in 96-well plates, and after 24 h of growth, increasing amounts of extract were added. Cells viability was assessed at three different incubation times (24, 48 and 72 h) by MTT method. The ratio between absorbance values of treated cells and those concerning untreated cells (positive control) indicates the rate of cell proliferation (in our case expressed as cell viability percentage). Apoptosis was detected by differential acridine orange/ethidium bromide (AO/EB) staining [36] on HeLa and CRL2130 cells by using a procedure described in [37].

### 4.8. Immunofluorescence Experiments

The effect of *A. annua* PCSL extract on stress granules recruitment was tested in HeLa cells. Briefly, 2 × 10^4^ HeLa cells were seeded on coverslips, cultured for 24 h and then treated at 37 °C for 1 h with two different dilutions of extract (1:10 and 1:100). The treatment of HeLa cells with 500 µM of sodium arsenite for 1 h at 37 °C was considered as a positive control of stress granules recruitment.

Cells were finally fixed in 4% paraformaldehyde for 15 min, permeabilized in 0.1% Triton-X100 for 5 min and blocked with 1% BSA (bovine serum albumin) for 20 min. Subsequently, cells were incubated with mouse anti-PABP (P6246-Sigma-Aldrich), diluted 1:750 in BSA 1% for 1 h, and then with the secondary antibody goat anti-Mouse (DyLight ^®^ 594 Conjugate). Nuclear staining of the cells was obtained by incubation with DAPI (Molecular Probes, Invitrogen, Italy) diluted 1:2000 in PBS for 5 min at room temperature. After washing, coverslips were mounted in Mowiol ^®^ 4–88 (Sigma Aldrich, Milan, Italy) on microscope slides. Confocal microscopy observations were performed using Zeiss Confocal Microscope LSM 900 and a 63× oil objective.

### 4.9. Real-Time qPCR Analysis

Expression of Bax gene, a pro-apoptotic marker, was evaluated by Real-Time qPCR in HeLa cells subjected to treatment with two alternative doses of PCSL extract. In brief, 3 × 10^5^ cells were plated, and after 24 h, exposed to PCLS extract (1:10 or 1:100) for 1 h at 37 °C. Total RNA was then extracted using TRIzol ™ Reagent (Invitrogen ™), according to the manufacturer’s instructions, quantified by Nanodrop 8000 (Thermo-Scientific) and finally retro-transcripted to cDNA with SuperScript ™ IV VILO ™ Master Mix (Invitrogen ™). RT-PCRq was performed by StepOnePlus ™ Real-Time PCR System (Applied Biosystems ™) using the primers listed in Table 1. Expression of Bax gene was normalized with respect to the housekeeping gene encoding GAPDH.

### 4.10. Statistical Analysis

Data were analysed with GraphPad Prism software (version 5.0-GraphPad Inc., San Diego, CA, USA) by using Student’s *t* test. A *p* value of 0.05 or less was considered statistically significant (* *p* < 0.05, ** *p* < 0.01, *** *p* < 0.001 or ****p < 0.0001).

## Figures and Tables

**Table 1:** Primer sequences of selected genes.

Scheme	Forward	Reverse
Bax [38]	5′-TGCTTCAGGGTTTCATCCAG-3′	5′ -GGCGGCAATCATCCTCTG-3′
GAPDH	5′-CACCACACTGAATCTCCCCT-3′	5′-TGGTTGAGCACAGGGTACTT- 3′

## Data Availability

Not applicable.
